# Dependency Links Can Hinder the Evolution of Cooperation in the Prisoner’s Dilemma Game on Lattices and Networks

**DOI:** 10.1371/journal.pone.0121508

**Published:** 2015-03-23

**Authors:** Xuwen Wang, Sen Nie, Binghong Wang

**Affiliations:** 1 Department of Modern Physics, University of Science and Technology of China, Hefei, Anhui, 230026, P. R. China; 2 College of Physics and Electronic Information Engineering, Wenzhou University, Wenzhou, Zhejiang, 325035, P. R. China; 3 School of Science, Southwest University of Science and Technology, Mianyang, Sichuan, 621010, P. R. China; Hong Kong Baptist University, CHINA

## Abstract

Networks with dependency links are more vulnerable when facing the attacks. Recent research also has demonstrated that the interdependent groups support the spreading of cooperation. We study the prisoner’s dilemma games on spatial networks with dependency links, in which a fraction of individual pairs is selected to depend on each other. The dependency individuals can gain an extra payoff whose value is between the payoff of mutual cooperation and the value of temptation to defect. Thus, this mechanism reflects that the dependency relation is stronger than the relation of ordinary mutual cooperation, but it is not large enough to cause the defection of the dependency pair. We show that the dependence of individuals hinders, promotes and never affects the cooperation on regular ring networks, square lattice, random and scale-free networks, respectively. The results for the square lattice and regular ring networks are demonstrated by the pair approximation.

## Introduction

Cooperation is a ubiquitous behavior that exists in the natural and social systems [1–3]. However, there are many social dilemmas, which prevent the cooperation among the selfish individuals, including the prisoner’s dilemma games [4–8], snowdrift games [9, 10] and public goods games [11–16]. If both players aim at maximizing their own payoff, then no player has an incentive to switch unilaterally from defection to cooperation. Thus, to maintain and promote the cooperation is a fundamental problem in social and biology science. In the original PDG, the individuals can choose one of two strategies, cooperation or defection, and they both receive payoff *R* upon mutual cooperation and *P* upon mutual defection. If one defects while the other cooperates, the cooperator receives *S* while the defector gets *T*. The ranking of the four payoff values is *T* > *R* > *P* > *S* and 2*R* > *T* + *S*. To promote and characterize the cooperation of real systems, the games on the structured populations have been widely described, such as cooperation on square-lattice [17–20], small-world [21–23], scale-free [24–26], and coevolving networks [27–29]. Moreover, many evolutionary rules that can affect the cooperation have also been introduced, including the reward [30, 31], punishment [32–35], diversity [14, 36, 37] and extortion strategy [38–40].

Recently, motivated by the fact that the networks are often coupled together, the cooperation on the interdependent networks has been also studied [41, 42]. For two interdependent networks, the individuals in one network are linked to ones in the other network, and it has been demonstrated that these networks can support the reciprocity of prisoner’s dilemma games and public goods games. Besides this, another type of dependency relation is built in one network which contains both connectivity and dependency links [43–45]. The former works mainly focused on how the interdependent structures affected the cooperation. While the consideration of repeated prisoner’s dilemma game is that most of us will interact with household members, colleagues, and other people in small-scale economic interactions again and again [1]. Hence, individuals will most likely behave differently when facing different interaction objects. Especially for kin relationship individual pairs, it is difficult for them to behave mutual defecting but they are always cooperating. Here, we introduce a dependency relation into the prisoner’s dilemma games to feature the interaction which is analogous to the kin relationship and other solid relations. The dependency individuals can gain an extra payoff, and this payoff is larger than the payoff of mutual cooperation *R* but is lower than the temptation to defect *T*. Hence, dependency relation is stronger than the ordinary mutual cooperation interaction, but not large enough to cause the defection. We find that the dependence of individuals hinders the cooperation on regular ring networks, while it slightly affects cooperation on random and scale-free networks. The only case that can promote the cooperation is the square lattice networks. In particular, below the half density of dependency links, the larger fraction of dependency pairs, the more disadvantage of cooperation for small average degree of regular ring networks.

The paper is organized as follows: Section 2 is the detailed description of the model. In Section 3, we present the simulation results. The Section 4 is the discussion.

## Model

Individuals locate on the networks, in which each one is linked with its nearest neighbours. Initially, each individual is designated either as a cooperator or defector with equal probability and *f* fraction of individual pairs is selected randomly to interdepend with each other. For *f* = 0 the model reduces to the original PDG, while the whole individuals are dependency pairs for the case of *f* = 0.5. The higher value of *f*, the larger fraction of individuals are dependents. For each Monte Carlo step, individual *x* plays the PDG with its nearest neighbors. Its payoff contains two portions: the ordinary payoff and the payoff offered by dependency individual, respectively. *P*_*x*_ is the sum of all the payoffs acquired from its nearest ordinary neighbours. However, if an individual is dependent with another one, then it can gain an extra payoff:

$$\begin{array}{c} P_{e}=1+\frac{b-1}{2}, \end{array}$$ (1)

In this, for simplicity but without loss of generality, the payoff matrix for the PDG are rescaled such that *R* = 1, *S* = 0, *T* = *b*, *P* = 0, where 1 < *b* ≤ 2 represents the advantage of defectors over cooperators. Equation 1 guarantees the extra payoff 1 < *P*_*e*_ < *b*. Then, the individual *x* adopts a randomly selected neighbor *y*’s strategy with the probability:

$$f_{x\to y}=max\{0,\frac{P_{t}(y)-P_{t}(x)}{\Theta k_{>}+P_{e}}\},$$ (2)

where *k*_>_ = *max*{*k*_*i*_, *k*_*j*_}, Θ = *T* − *S* in the PDG and *P*_*t*_(*y*) is the total payoff of individual *y*. We set network size *N* = 10000 for all simulations. We study the prisoner’s dilemma games on the regular ring networks, square lattice, random and scale-free networks. The regular ring networks and square lattice networks are used the periodic boundary conditions. The scale-free networks are generated by the B*á*rabasi-Albert model [46, 47].

## Results

**Fig 1:**
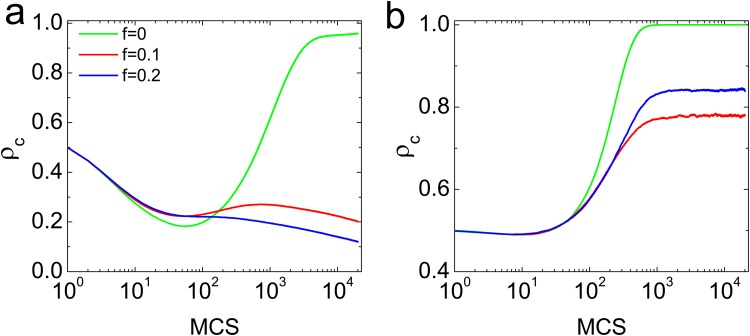
(Color online) Time evolving of cooperation level on the regular ring networks for *b* = 1.05 (a) The regular ring networks with *k* = 4 and (b) The regular ring networks with *k* = 8. Each data point is an average of 100 independent realizations.

Firstly, we investigate how the dependency links affect the time evolution of cooperation level on regular ring networks for different dependency density *f*, which is shown in Fig. 1. To the original PDG with *f* = 0 and average degree < *k* > = 4, the cooperation level decreases rapidly due to the advantage of defection at the beginning of evolution. While the cooperators can survive in the structured population by forming the clusters, which in turn to increase the cooperation level in the latter steps. This is actually consistent with the fact that the structured population facilitates network reciprocity [18, 48]. But interestingly, the incorporating of dependency links makes the gradual increasing of cooperation level impossible, then the cooperation level finally arrives at a relatively lower value. In this sense, the dependency link weakens the effect of network reciprocity. For the case of < *k* > = 8, the dependency relation also hinders the cooperation. In particular, the higher density of dependency links, the greater resistance to cooperation behavior.

**Fig 2:**
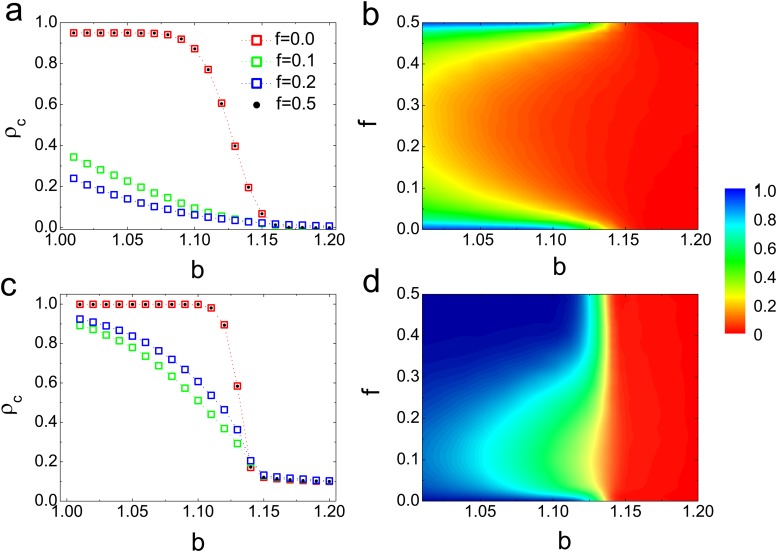
(Color online) Cooperation level as a function of temptation to defect *b* for different dependency densities (a) The regular ring networks with *k* = 4 and (c) The regular ring networks with *k* = 8. (b) and (d) are cooperation level on the *b* − *f* parameter plane for *k* = 4 and 8, respectively. Each data point is an average of 500 independent realizations.

To intuitively show the effect of dependency links on the cooperation for regular lattice networks, we examine the cooperation level as a function of temptation to defect *b* and dependency density *f* for different average degrees. Fig. 2 depicts that the dependence of individuals hinders the cooperation for both two average degrees which are irrespective with the value of *b*. To summarize, there are three distinct phenomenons for two average degrees: (i) The cooperation level is symmetric with *f* = 0.25 for < *k* > = 4, while it has no obvious symmetry of results for < *k* > = 8. (ii) As *f* < 0.25, the higher density of dependency links, the lower cooperation level for < *k* > = 4, which is totally different from the result for < *k* > = 8. (iii) The dependence of links affects the cooperation level greater for small average degree than that for large ones. Especially, the cooperation level has a larger downtrend as intermediate *b* for < *k* > = 4. If each individual can gain an extra payoff from its interdependent neighbour, then the strategy updating in Equation 2 hold unchanged. As a consequence, the density of cooperators for *f* = 0 is the same as *f* = 0.5.

**Fig 3:**
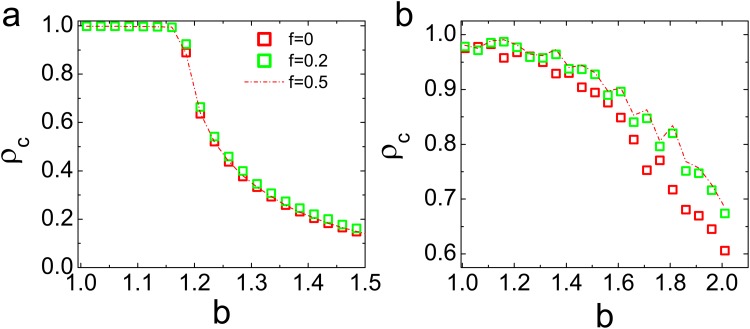
(Color online) Cooperation level as a function of temptation to defect *b* (a) The random networks with < *k* > = 4 and (b) The scale-free networks with < *k* > = 4. Each data point is an average of 500 independent realizations.

The network’s structure plays an important role in the evolutionary games. It has been proved that the scale-free networks can greatly support the cooperation due to the existence of hub nodes [24]. Hence, it is necessary for us to examine the cooperation level as a function of *b* for the random networks and scale-free networks with dependency links. Unlike to the results of regular ring networks in Fig. 2, the fraction of dependency links no longer affects the cooperation as shown in Fig. 3. Indeed, the regular ring networks and scale-free networks are two examples of the simple degree-homogeneous and highly degree-heterogeneous graphs, respectively [25]. Above results demonstrate that the dependency relation can only affect the evolution of cooperation on the degree-homogeneous networks. On the contrary, the extra payoff for the individuals cannot change the tendency of strategy adoption. As an example, examining the case of a *C* − *D* individual pair in the competing domains. The cooperator surrounded by its neighbours may have a payoff *P*_*C*_ = 3(< *k* > = 4), while the defector may get a payoff *P*_*D*_ = 2*b* from its ordinary neighbours. However, the payoff of defector may be larger than that of cooperator when the defector can gain an extra payoff from its dependent neighbour, and then the strategy adoption *D* → *C* turns into *C* → *D*. This transformation induces the increment of cooperation level impossible, which is consistent with the simulation result in Fig. 1. However, the situation of the degree-heterogeneous networks can be significantly different. The larger differences of *k*_*C*_ − *k*_*D*_ and *P*_*C*_ − *P*_*D*_ make the extra payoff *P*_*e*_ cannot be large enough to change the transformation from *D* → *C* into *C* → *D*. We can also observe the similar results for large *b* in Fig. 2(a) and Fig. 2(c).

**Fig 4:**
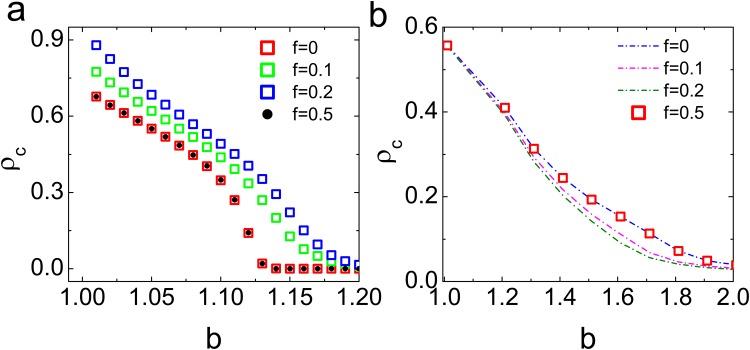
(Color online) Cooperation level as a function of temptation to defect *b* (a) The square lattice with *k* = 4 and (b) The curves obtained by the pair approximation approach for the regular networks with *k* = 4. Each data point in (a) is an average of 500 independent realizations.

Next, we examine the results on the square lattice networks, which are also degree-homogeneous graphs. Very interestingly in Fig. 4(a), we observe that the dependency relation can slightly support the cooperation, which is contrary to the results of regular ring networks even if both of them have the same degree distribution. The higher density of dependency links, the more contribution to cooperation. Even if two different types of networks have the exact same degree distribution, but their clustering coefficients are different. The square lattice network has no triangles, hence its clustering coefficient *C* = 0 [49]. Nevertheless, the regular ring network has *C* = 1/2. This difference may cause the entirely different phenomenons of cooperation level on the regular ring networks and square lattice networks with dependency links.

Finally, We apply the pair approximation method [50, 51] to qualitatively predict cooperation behavior by incorporating the dependency relation. For the focal sites *A* and *B*, the strategy of the player *A* is updated by comparing his performance to a randomly chosen neighbour *B*. The payoffs *P*_*A*_ and *P*_*B*_ of *A* and *B* are determined by the total payoffs from their neighbours *x*, *y*, *z*, *B* and *u*, *v*, *w*, *A*, respectively. The pair approximation is completed by determining the evolution of the pair configuration probability, that is, the probability that the pair *p*_*A*,*B*_ becomes *p*_*B*,*B*_:

$$\begin{array}{c} p_{A,B\to B,B}=\sum_{x,y,z}\sum_{u,v,w}f\left(P_{B}-P_{A}\right)\times \frac{p_{x,A}p_{y,A}p_{z,A}p_{A,B}p_{u,B}p_{v,B}p_{w,B}}{p_{A}^{3}p_{B}^{3}}. \end{array}$$ (3)

The evolving of cooperation level can be obtained by the following ordinary differential equations:

$$\begin{array}{cc} {\dot{p}}_{c,c}= & \sum_{xyz}\left[n_{c}\left(x,y,z\right)+1\right]p_{d,x}p_{d,y}p_{d,z}\\ & \times \sum_{u,v,w}p_{c,u}p_{c,v}p_{c,w}f\left(P_{c}\left(u,v,w,\Omega_{u},\Omega_{v},\Omega_{w}\right)-P_{d}\left(x,y,z,\Omega_{x},\Omega_{y},\Omega_{z}\right)\right)\\ & -\sum_{xyz}n_{c}\left(x,y,z\right)p_{c,x}p_{c,y}p_{c,z}\\ & \times \sum_{u,v,w}p_{d,u}p_{d,v}p_{d,w}f\left(P_{d}\left(u,v,w,\Omega_{u},\Omega_{v},\Omega_{w}\right)-P_{c}\left(x,y,z,\Omega_{x},\Omega_{y},\Omega_{z}\right)\right), \end{array}$$ (4)

$$\begin{array}{cc} {\dot{p}}_{c,d}= & \sum_{xyz}\left[1-n_{c}\left(x,y,z\right)\right]p_{d,x}p_{d,y}p_{d,z}\\ & \times \sum_{u,v,w}p_{c,u}p_{c,v}p_{c,w}f\left(P_{c}\left(u,v,w,\Omega_{u},\Omega_{v},\Omega_{w}\right)-P_{d}\left(x,y,z,\Omega_{x},\Omega_{y},\Omega_{z}\right)\right)\\ & -\sum_{xyz}\left[2-n_{c}\left(x,y,z\right)\right]p_{c,x}p_{c,y}p_{c,z}\\ & \times \sum_{u,v,w}p_{d,u}p_{d,v}p_{d,w}f\left(P_{d}\left(u,v,w,\Omega_{u},\Omega_{v},\Omega_{w}\right)-P_{c}\left(x,y,z,\Omega_{x},\Omega_{y},\Omega_{z}\right)\right). \end{array}$$ (5)

Where *n*_*c*_(*x*, *y*, *z*) is the number of cooperators among the neighbours *x*, *y*, *z*, and *P*_*c*_(*x*, *y*, *z*) and *P*_*d*_(*x*, *y*, *z*) specify the payoffs of a cooperator(defector) interacting with the neighbours *x*, *y*, *z* plus a defector(cooperator). In Equation 4 and 5, *f*(*P*_*c*_ − *P*_*d*_) and *f*(*P*_*d*_ − *P*_*c*_) are the expectation transition probability. The equilibrium values are obtained by numerical integration and the common factor $2p_{\left(c,d)/(p_{c}^{3}p_{d}^{3}\right)}$ is omitted. The fraction of cooperators in the whole population is calculated by *p*_*c*_ = *p*_*c*,*c*_ + *p*_*c*,*d*_.

The prediction result in Fig. 4(b) shows that the dependency relation hinders the cooperation level and the higher density of dependency links induces the lower cooperation level. In addition, it also depicts that the cooperation level is symmetric with *f* = 0.25. Because the predictions of the pair approximation for any two regular graphs with the same average degree *k* are exactly the same [25], then the Fig. 4(b) only correctly presents the evolving tendency of cooperation level for regular ring networks. Additionally, it significantly over-estimates the benefits of population structures, which is similar to the results in Ref [50].

## Discussion

Many natural and man-made systems can be modeled as networks. Generally, the networks are interdependent with each other. The nodes in one network may depend on others in another network, then the failure of a node also causes the failure of its interdependent node [52–54]. In addition, a single network can also contain both connectivity and dependency links together [43, 44]. The dependency relation is also used to study the cooperation behaviors, and it has been demonstrated that interdependent networks can spread the cooperation [41, 42]. Motivated by the fact that individuals behave differently when facing the various interaction objects, we introduce a dependency relation into the spatial prisoner’s dilemma games to examine whether the dependency links can also promote the cooperation as the same as former researches. Specifically, a fraction *f* of individual pairs is selected randomly to depend with each other. This dependency relation can bring an extra payoff to the individual which is between the payoff of mutual cooperation *R* and temptation to defect *T*. The motivation of this setup is that the dependency relation ought to be stronger than ordinary cooperation but it cannot induce the defection between the dependent individuals.

It is found that the dependency relation hinders the cooperation on the regular ring networks, while it cannot affect the cooperation on random networks and scale-free networks. The only case that it can promote is the square lattice networks. Whether the dependency relation can affect the cooperation is determined by the homogeneous or heterogeneous of degree. For degree-heterogeneous networks, the extra payoff obtained from the individual’s dependency neighbour cannot change the transformation of the strategy adoption, hence, the dependency relation has no effects on the evolving of cooperation. Our result indicates that the dependency links cannot greatly promote the cooperation of spatial prisoner’s dilemma games, which is also different from the recent research.
